# Food Poisoning

**Published:** 1921-10

**Authors:** 


### FOOD POISONING.

A LETTER has recently been issued by the Ministry of Health, which includes a memorandum on the collection and transmission of samples in cases of food poisoning. Dr. W. G. Savage, the Medical Officer of Health for Somerset (an article by whom we publish in this number) will be in charge of the investigation. His notes on the collection of samples are as follows :—

(1) *Any Portions of the Supposed Peccant Food.*—These should be actual portions remaining over from the food which caused the illness. Even small scraps, remains in tins (if a tinned food) are better than nothing. Samples of food of similar origin are usually of little assistance but occasionally are useful.

(2) *Post-mortem Materials from Fatal Cases.*—The spleen, pieces of liver, piece of small intestine, kidney, are the most useful. The stomach (ligatured) with unopened contents is valuable if metallic poisoning is suspected, but not of much use otherwise.

(3) *Pathological Material from the Cases.*—From all cases, and especially when (1) is not available, and (2) only problematical, endeavours should be made to collect and transmit material from the actual sufferers while in the acute stages. The samples should be excreta samples and vomit. Of these, excreta samples are both the easier to obtain and the more valuable.

(4) *Samples of Blood for Serological Examination from the Sufferers.*—There is no urgency about these samples since any serological reactions persist for many weeks, while in any case they will not develop until a week or more after the onset. It is better, therefore, to wait to collect these samples until after the bacteriologist has the other samples, and also the preliminary information as to the outbreak, so that he can indicate exactly what he requires.

### TRANSMISSION.

Outfits are available at the Bristol Laboratory, but usually there will not be time to send for these, and the transmittors must use their laboratory outfits or an emergency one. Excreta specimens can be sent in clean wide-mouth bottles. The food can be put in a clean tobacco tin. The organs from fatal cases should each be wrapped in a piece of clean cloth wetted and wrung out (only damp, therefore) with 1 in 500 corrosive sublimate solution. All the organs can be placed so wrapped in one large tobacco or other tin. Outfits for blood samples will be sent from the laboratory.

If possible, the samples should be sent packed in ice. An impromptu ice box can be made by using a fairly large biscuit tin, and packing the tins and bottles in one side of this and filling the other part with dry ice in small pieces. The tins and bottles must be secured from rolling about when the ice melts, by means of cloths and wood partition, or other device. The biscuit box should be packed in a wood box with at least two inches everywhere of sawdust between the two boxes. If ice is not procurable the samples must be sent without it rather than delay.

Samples should be sent by passenger train to Mr. P. Bruce White, Pathology Department, University of Bristol, who should be informed by telegram of their despatch, and of the probable time of arrival.

---

THE London County Council decided recently that for medical or dental treatment of school children the charge should be one shilling for each period of six months, with free treatment for the first fortnight.

### NURSES AND UNEMPLOYMENT INSURANCE.

THE College of Nursing is organising a deputation to the Ministry of Labour on the vexed question of the inclusion of nurses in the Unemployment Insurance Act. Credit is due to the Council of the College for its persistence in this matter throughout; had the nursing profession as individuals taken the trouble to express in writing at the proper time the wish which, practically without exception, each nurse or probationer cherishes, it is more than likely that nurses would have been excluded, or that an Amending Act would have been passed. Now that another opportunity is given to them to state their desire for exclusion, we hope every sister, nurse, or probationer will take advantage of it and send her signature to the College of Nursing before October 31, so that the deputation may have behind it a strong body of nursing opinion. The strength of the case for exclusion is that nurses as a class do not suffer from unemployment except through ill-health or advancing years. In the former case they are not eligible for unemployment insurance, and in the latter superannuation payments are the right and appropriate means of dealing with the situation. If, instead of employers of nurses and their employees paying into the unemployment-insurance fund, they were to contribute to a superannuation scheme such as that the College of Nursing is at present working out, nurses would be benefited in a much more practical and effective manner.

---

### CLOSED BEDS AND MEDICAL SCHOOLS.

THE medical schools, as everyone knows, have received a large entry of students since the war, and it is one of the ironies of the situation that the hospitals where the students gain their clinical experience should have had to close beds at this particular time. The Faculty of Medicine of the University of Birmingham has addressed a letter of regret to the Chairman of the General Hospital, Birmingham, where fifty beds were lately closed. The letter, signed by the Vice-Chancellor, Sir Gilbert Barling, Professor Evans Robertson (Principal), and Mr. W. F. Haslam (Dean of the Medical Faculty), says: " Those responsible for the clinical teaching have had to draw up a scheme so that each medical student may have a proper amount of clinical material allotted to him under the supervision of his Chief, and sufficient time given him for instruction in the same. The scheme, which entails extra work on the staff, has been prepared with considerable trouble and on the assumption that the full number of beds would be available. The reduction of this number by fifty will render it more difficult to carry out." The signatories also point out that many of the students will ultimately settle in the neighbourhood. The hospital board can only share the regret of the medical faculty, and in reply states that though the special efforts made to increase the hospital's income " are meeting with a certain measure of success," it is impossible to say when the wards can be reopened.

